# Occupational Disparities in Lifestyle Behaviors and Adiposity Levels Among Working Women in Peru: A Pooled Repeated Cross-Sectional Analysis of 10 Rounds of a National Health Survey

**DOI:** 10.3390/healthcare14121763

**Published:** 2026-06-18

**Authors:** Víctor Juan Vera-Ponce, Jhosmer Ballena-Caicedo, Fiorella E. Zuzunaga-Montoya

**Affiliations:** Facultad de Medicina (FAMED), Universidad Nacional Toribio Rodríguez de Mendoza de Amazonas (UNTRM), Calle Higos Urco No. 342-350-356, Chachapoyas 01001, Amazonas, Peru; 7330178022@untrm.edu.pe (J.B.-C.); fiorella.zuzunaga@untrm.edu.pe (F.E.Z.-M.)

**Keywords:** occupational groups, women, working, health behavior, obesity, cross-sectional studies, body mass index, waist circumference, health disparities, Peru

## Abstract

**Background/Objectives:** Occupation shapes time use, physical demands, stress, and access to health resources, yet it remains an understudied axis of inequality among working women in low- and middle-income countries. This study assessed occupational-group disparities in lifestyle behaviors and adiposity levels among Peruvian working women. **Methods:** We conducted a pooled repeated cross-sectional analysis of ten Peruvian DHS/ENDES rounds from 2014–2019 and 2021–2024 among working women aged 18–49 years. The exposure was standardized occupational group, using professional/technical/managerial workers as the reference. Outcomes included five lifestyle behaviors and four adiposity indicators. Crude models estimated descriptive prevalence ratios (PRs) or beta coefficients; secondary adjusted models included age group, survey year, education, wealth, residence, natural region, and marital status. **Results:** A total of 40,726 women were included. Agricultural workers showed lower crude prevalences of almost-daily television viewing (PR 0.49; 95% CI 0.47–0.52), current smoking (PR 0.14; 95% CI 0.10–0.19), current alcohol use (PR 0.39; 95% CI 0.36–0.42), and heavy alcohol use (PR 0.17; 95% CI 0.12–0.27); these contrasts attenuated but generally persisted after adjustment. Insufficient fruit and vegetable intake exceeded 87% in all groups. Sales, domestic/household, services, and skilled manual workers had higher adjusted obesity than the reference group, with adjusted PRs ranging from 1.22 to 1.35. **Conclusions:** Occupation identifies relevant heterogeneity in lifestyle behaviors and adiposity levels among Peruvian working women. Lifestyle and adiposity profiles did not follow a simple social gradient, supporting occupation-specific strategies for noncommunicable disease prevention.

## 1. Introduction

Noncommunicable diseases and adiposity continue to expand globally, with a growing burden among women of working age and an increasingly important role of central adiposity as a marker of cardiometabolic risk [1,2,3,4]. In this study, central adiposity was operationalized through waist circumference (WC) and abdominal obesity (AO), defined as WC ≥ 88 cm in women. At the same time, physical inactivity and sedentary behavior remain highly prevalent and are unevenly distributed across populations and living environments [2,3]. In this context, occupation matters not only as a socioeconomic marker but also as a daily structure that shapes time use, physical demands, autonomy, stress, screen exposure, eating routines, and social norms related to tobacco and alcohol use.

Occupation can be understood as a structural determinant because it organizes exposure to material resources, time constraints, physical workload, job control, psychosocial demands, and social protection. Among women, these occupational conditions may intersect with gendered labor-market segmentation and unpaid domestic or caregiving responsibilities. Therefore, occupational group is not merely an individual socioeconomic label; it is a population-level marker of how work structures opportunities and constraints for health-related behaviors and adiposity.

Available evidence suggests that the relationship between work and health is neither linear nor uniform. Prolonged sedentary time is associated with adverse outcomes, although part of that risk may be modified by higher levels of total physical activity [5]. In addition, physical and psychosocial work-related risk factors tend to cluster with obesity, smoking, and leisure-time physical inactivity, particularly in occupations with less control and greater cumulative burden [6]. However, a substantial share of this literature has examined workers in general, with less attention to how these inequalities manifest specifically among women.

This omission is important. The growing participation of women in the labor market has occurred alongside persistent gender-based occupational segmentation and the coexistence of paid work, domestic responsibilities, and unpaid caregiving. A recent systematic review of working women documented high prevalences of overweight/obesity, low physical activity, and unhealthy diet, and identified long working hours, double burden, and job stress as recurrent correlates [7]. Studies in Brazilian shift workers, prospective cohorts in Finland, and occupational analyses in the Southern Cone of Latin America also suggest that work organization may shape both behaviors and cardiometabolic risk, but the generalizability of these findings remains limited [8,9,10].

Evidence on diet by occupation among employed women also suggests relevant heterogeneity, although it often comes from specific cohorts and uses populations that are not directly comparable [11]. In Peru, despite the magnitude of the nutrition transition and the sustained growth of noncommunicable diseases, national analyses are lacking that jointly assess lifestyle and anthropometric outcomes by occupational group among working women. In this study, we sought to fill this gap by analyzing repeated DHS rounds to evaluate occupational disparities in health-related behaviors and adiposity among Peruvian working women.

## 2. Materials and Methods

No chemicals, reagents, devices, instruments, commercial cell lines, biological samples, or laboratory materials were used because this study was a secondary analysis of publicly available anonymized survey microdata.

### 2.1. Study Design

An observational analytical cross-sectional study was conducted based on repeated cross-sectional series from the Peruvian Demographic and Family Health Survey (DHS). Publicly available anonymized secondary microdata from the 2014–2019 and 2021–2024 rounds (ten rounds in total) were used; this period represented the span during which the prespecified combination of exposure, outcomes, and covariates could be harmonized consistently in the analytic file. The 2020 round was excluded a priori because the COVID-19 pandemic introduced operational disruptions in fieldwork and generated a substantive population-level shock to work, mobility, health behaviors, and anthropometric measurement conditions. Therefore, including 2020 would not provide a neutral sensitivity analysis but would instead mix regular survey years with an atypical pandemic round of lower comparability. The study was reported following the STROBE guideline for cross-sectional observational studies (Table S1) [12].

### 2.2. Data Source

The DHS is a continuous population-based survey conducted by the National Institute of Statistics and Informatics of Peru, designed to generate estimates with national and geographic-domain representativeness. Its sampling design is complex, probabilistic, stratified, and multistage. For the present study, two survey components were used: the women’s individual questionnaire, from which occupation and part of the sociodemographic covariates were obtained, and the health questionnaire administered to one person aged 18 years or older selected per household, from which health behaviors and anthropometric measurements were obtained [13].

### 2.3. Study Population and Eligibility Criteria

The target population consisted of women aged 18–49 years who were usual residents of private households in Peru. In accordance with the DHS/ENDES sampling framework, private households refer to non-institutional dwellings selected in urban and rural areas, including usual residents who stayed in the household the night before the interview. The analytic file required linkable information between the women’s individual questionnaire and the health module. The former provided occupation and sociodemographic variables, whereas the latter provided health behaviors and anthropometric measurements for the selected household member. Person-level linkage was performed using the household identifier and respondent line number; when validation variables were available, only records in which the woman interviewed in the individual questionnaire matched the person selected for the health module were retained. This restriction was necessary because the main exposure came from the individual questionnaire and the outcomes from the health module.

The main analysis was restricted to women who reported currently working and had a coded occupational group. The “not working” category was not included as a main exposure level because it groups substantively heterogeneous situations—unemployment, unpaid domestic work, studying, temporary incapacity, or others—that do not represent a single interpretable type of occupational insertion. For this reason, comparing working and non-working women was not considered a valid occupational contrast in the present study; such an analysis would address labor-force participation rather than occupational-group disparities among workers. For behavioral analyses, participants with valid information on the corresponding outcome were included. Pregnant women were excluded from anthropometric analyses because BMI and WC are not directly interpretable as adiposity indicators during pregnancy. For the main anthropometric analyses, valid and plausible weight and height information was required so that the BMI and obesity outcomes would be estimated on the same analytic sample and be directly comparable. For central adiposity analyses, valid and plausible information on WC was additionally required.

### 2.4. Main Exposure

The main exposure was the respondent’s occupational group, derived from the DHS standardized occupation classification. The active employment categories were retained: professional/technical/managerial, clerical/administrative, sales, self-employed agriculture, domestic/household work, services, skilled manual, and unskilled manual. The professional/technical/managerial group was defined a priori as the reference category, not because a causal protective relationship was assumed, but because it represented higher-skilled nonmanual occupations, had sufficient sample size, and provided an interpretively useful contrast with the remaining categories. This decision sets the comparison point of the estimates but does not alter the model’s overall fit. Because the objective was to provide interpretable occupational contrasts rather than all possible pairwise comparisons, no exhaustive pairwise comparison matrix was generated.

### 2.5. Outcomes

Two families of outcomes were evaluated. Lifestyle behaviors comprised five binary variables: watching television almost every day, current smoking, current alcohol use, heavy alcohol use, and insufficient fruit and vegetable intake, defined as fewer than five servings per day. All were analyzed as binary variables. The main anthropometric outcomes were body mass index (BMI, kg/m^2^) as a continuous variable and general obesity (BMI ≥ 30 kg/m^2^) as a binary variable; waist circumference (WC) and abdominal obesity (AO) (WC ≥ 88 cm) were also incorporated. A composite “lifestyle” index was not constructed because this would have required assigning arbitrary weights to behaviors with different frequencies, epidemiologic meanings, and possible causal directions. Instead, each outcome was analyzed separately to preserve clinical and public health interpretability.

### 2.6. Covariates

Sociodemographic covariates were selected with the purpose of providing comparative characterization across occupational groups. These included age group (18–24, 25–34, and 35–49 years); educational level (no formal education, primary, secondary, higher); wealth quintile (poorest, poorer, middle, richer, richest), derived from the DHS wealth index; area of residence (urban, rural); natural region (Metropolitan Lima, Coast excluding Lima, Highlands, Jungle); marital status (never married, married/cohabiting, formerly married); and survey year (2014, 2015, 2016, 2017, 2018, 2019, 2021, 2022, 2023, and 2024), treated as a categorical variable.

Age was described in the three prespecified categories because the eligible age range was relatively narrow, because work-life stages within that range are epidemiologically distinguishable—with different profiles of occupational insertion, domestic burden, and cardiometabolic risk across the 18–24, 25–34, and 35–49-year groups—and because this parameterization avoids imposing a linear or otherwise arbitrary functional form. Although the DHS includes information on health insurance coverage, this variable was not incorporated into the main characterization because, in a population of working women, type of insurance may partly be a consequence of labor-market insertion, and its inclusion could artificially attenuate the occupational contrasts of interest.

### 2.7. Data Preparation and Cleaning

Data were cleaned using prespecified consistency and plausibility rules. For anthropometric measures, only plausible values for weight (20–250 kg), height (120–210 cm), and WC (45–180 cm) were retained; values outside these ranges, as well as special codes for nonresponse or nonmeasurement, were treated as missing. Subsequently, BMI values outside the plausible range (12–70 kg/m^2^) were also excluded. Categorical variables were harmonized across rounds before annual pooling.

Educational level required a special harmonization process. Variable QS25N (highest level of schooling completed, health module) was used as the primary source; however, in the 2015–2018 rounds, this variable had missing values for a very high proportion of observations because of differences in the administration of the health module in those rounds. To complete the missing information in these four rounds, variable V106 (educational level from the household questionnaire), available with complete coverage in all rounds, was used as a backup source. Before combining both sources, coding concordance was verified in rounds where both variables were simultaneously available: 0 = no education, 1 = primary, 2 = secondary, 3 = higher.

### 2.8. Statistical Analysis

All analyses incorporated DHS survey weights. For the combined file, the health-module weight of each round was rescaled by dividing it by the number of included rounds, so that the pooled file represented the average population structure of the analyzed period rather than the arithmetic sum of annual populations. Descriptive estimates were summarized as unweighted absolute frequencies and weighted proportions or means. Where appropriate, 95% confidence intervals were calculated for descriptive estimates and figures.

It is important to note that weight and height measurements were consistently available from 2014 onward, which allowed estimation of body mass index and general obesity across the full set of rounds included in the main analysis. In contrast, waist circumference was only available from 2018 onward, so analyses of mean waist circumference and abdominal obesity were conducted in a subsample restricted to the 2018–2019 and 2021–2024 rounds. This restriction affected only waist circumference-based outcomes and did not modify the sample used for lifestyle analyses, which remained based on the widest available time series. Consequently, central adiposity results should be interpreted as a subanalysis with more limited temporal coverage than the main analysis, although the retained sample size remained large and sufficient to obtain estimates with acceptable precision.

Behavioral outcomes and binary anthropometric outcomes were modeled using modified Poisson regression with a log link, survey weighting, and robust variance clustered at the primary sampling unit level; results were expressed as prevalence ratios (PRs) with 95% confidence intervals. For BMI, weighted linear models were used with the same robust variance structure, and beta coefficients (β) with 95% confidence intervals were reported.

Crude models were retained as the primary estimates. This decision is consistent with the study objective of describing the observed distribution of health behaviors and adiposity across occupational groups, rather than establishing whether the type of work directly causes these outcomes. Attributing a direct causal relationship between occupation and lifestyle behaviors would require assumptions that cross-sectional data cannot support—among them, that the observed differences do not reflect selection, pre-employment trajectories, or shared structural factors. Accordingly, the primary associations should be interpreted as observed descriptive contrasts, not as causal effects of work on health.

As a secondary sensitivity analysis, adjusted models were estimated, including age group, survey year, educational level, wealth quintile, area of residence, natural region, and marital status. These models were not interpreted as causal estimates of the direct effect of occupation, because several covariates may be structurally related to occupational insertion. Instead, they were used to assess whether the crude occupational contrasts were largely explained by sociodemographic composition or whether they persisted after accounting for major observed population characteristics.

As an exploratory temporal analysis, survey years were grouped into three periods—2014–2016, 2017–2019, and 2021–2024—and occupation-by-period interactions were tested for selected outcomes with sufficient event frequency. For WC-based outcomes, the periods were 2018–2019 and 2021–2024 because WC was available only from 2018 onward. This analysis was intended to describe possible temporal heterogeneity in occupational disparities rather than to estimate causal time trends.

Missing data were handled through complete-case analysis at the level of each outcome family. Multiple imputation was not applied because missing anthropometric data primarily corresponded to measurements not obtained in the field rather than to partially missing information that could be recovered with high credibility from other variables; additionally, the proportion of missing data in the main covariates was low after harmonization. As a missing-data sensitivity assessment, women included in and excluded from the BMI and WC analytic samples were compared according to key sociodemographic and occupational characteristics. Given that outcomes were defined a priori and represented conceptually distinct domains, no formal correction for multiple comparisons was applied; interpretation focused on the magnitude, precision, and coherence of the estimates rather than on a dichotomous reading of the *p* value.

Data cleaning and harmonization were conducted in Stata, version 18.0 (StataCorp LLC, College Station, TX, USA). Analytical tables, regression models, and figures were generated in Python, version 3.12.3 (Python Software Foundation, Beaverton, OR, USA), using pandas version 2.2.3, NumPy version 2.1.2, statsmodels version 0.14.4, and Matplotlib version 3.9.2. All statistical tests were two-sided and accompanied by 95% confidence intervals.

### 2.9. Ethical Considerations

The study was based exclusively on anonymized public-access secondary microdata. At no stage of the analysis were direct personal identifiers available. The original DHS/ENDES data collection was conducted by the survey-producing agency under standardized field procedures for respondent consent and refusal recording before interviews and health measurements. Public-use datasets are distributed without direct identifiers, and the present analysis used only de-identified records available through the official microdata repository. Because this was a secondary analysis of public, anonymized data, no additional approval from an institutional ethics committee was required [14]. All procedures were conducted in accordance with the ethical principles established in the Declaration of Helsinki and its subsequent revisions.

## 3. Results

### 3.1. Analytic Sample Derivation

Behavioral analyses used DHS waves from 2014 to 2019 and 2021 to 2024 (10 rounds), excluding 2020 because of the operational interruption during the COVID-19 pandemic and the substantive non-comparability of pandemic-period work and behavioral conditions. Educational level was harmonized using QS25N as the primary source and V106 as a backup for the 2015–2018 waves. The primary analytic sample for behavioral outcomes comprised 40,726 working women aged 18–49 years. For adiposity levels, the main analysis was based on BMI, available in all waves after excluding pregnant women and observations with invalid or missing anthropometric information (*n* = 39,212). Because WC in the DHS is only available from 2018 onward, a subsample was used when it was included (six rounds; *n* = 21,216). The detailed analytic sample derivation, including exclusions by outcome family, is shown in Figure S1.

### 3.2. Sample Characteristics

Weighted characteristics of the analytic sample overall and by occupational group are presented in Table 1. The overall weighted mean age was 31.7 years (SD 9.7). The three largest groups were sales (*n* = 12,188; 29.9%), self-employed agriculture (*n* = 9805; 24.1%), and professional/technical/managerial (*n* = 6876; 16.9%). Professional/technical/managerial workers had the highest levels of higher education (84.4%), wealth (44.8% in the richest quintile), and urban residence (95.1%), as well as the highest mean age (32.9 years). Agricultural workers showed the opposite socioeconomic profile: 66.6% were in the poorest quintile, 42.8% had primary education, and 75.8% lived in rural areas.

### 3.3. Lifestyle Behaviors

**Figure 1:**
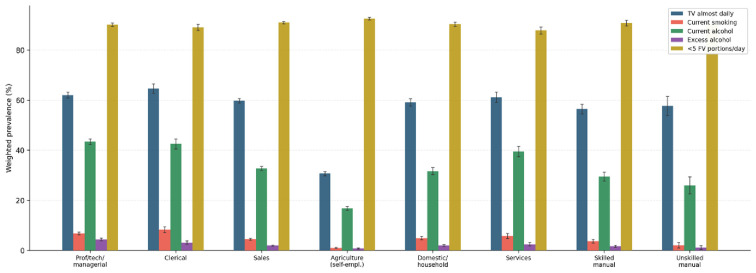
Weighted prevalence of behavioral outcomes by occupational group.

Weighted prevalences of lifestyle behaviors are illustrated in Figure 1 (numerical data in Table S2). Watching television almost daily was the most prevalent behavior (55.4% overall), with the lowest prevalence in agriculture (30.7%) and the highest in clerical/administrative occupations (64.6%). Current alcohol use affected 32.8% of the sample: it was highest in professional/technical/managerial workers (43.4%) and clerical/administrative workers (42.5%), and markedly lower in agriculture (16.8%). Current smoking was low in all groups (4.6% overall), ranging from 1.0% in agriculture to 8.3% in clerical/administrative occupations. Heavy alcohol use was infrequent (2.3% overall). Insufficient fruit and vegetable intake was uniformly high across all groups (>87%).

**Figure 2:**
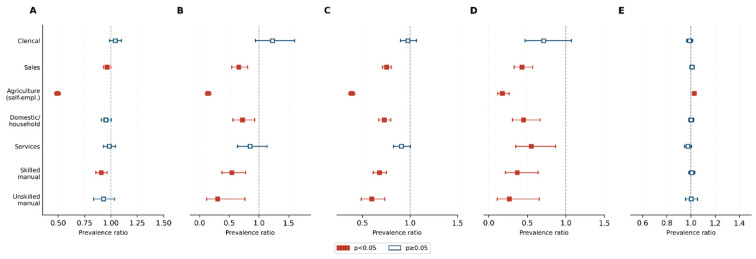
Crude prevalence ratios for behavioral outcomes by occupational group. Panels: (**A**) TV almost daily; (**B**) Current smoking; (**C**) Current alcohol use; (**D**) Heavy alcohol use; (**E**) <5 FV servings/day. Unadjusted models. Red square: *p* < 0.05; open blue square: *p* ≥ 0.05.

Crude prevalence ratios (PRs) are presented in Figure 2 (numeric values in Table S3). Agricultural workers had the lowest PRs for television viewing (PR 0.49; 95% CI 0.47–0.52), smoking (PR 0.14; 95% CI 0.10–0.19), and current alcohol use (PR 0.39; 95% CI 0.36–0.42). Heavy alcohol use was lower in sales (PR 0.43; 95% CI 0.33–0.57), domestic/household work (PR 0.45; 95% CI 0.31–0.67), and agriculture (PR 0.17; 95% CI 0.12–0.27). No relevant occupational differences were observed for insufficient fruit and vegetable intake. Models for daily smoking and alcohol use disaggregated by pattern are presented in Figure S3 and Table S4.

### 3.4. Adiposity Levels

**Figure 3:**
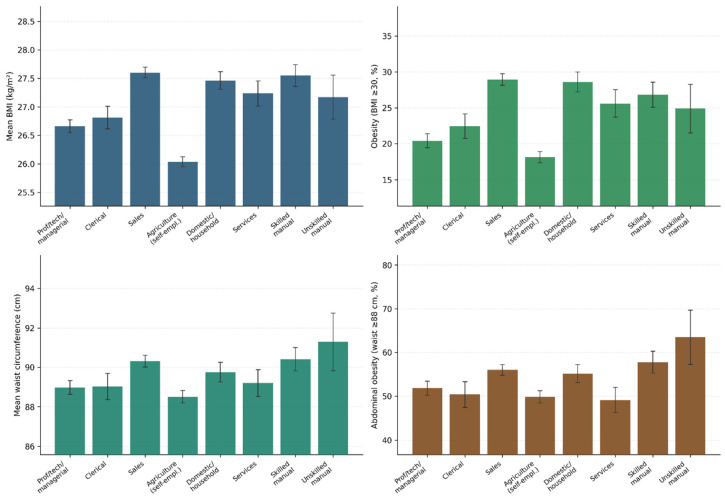
Weighted adiposity levels by occupational group. Upper panels: mean BMI and obesity prevalence. Lower panels: mean WC and AO prevalence.

Weighted adiposity levels are illustrated in Figure 3. The upper panels show mean BMI and obesity prevalence (main analysis; 10 waves, *n* = 39,212); the lower panels show mean WC and AO prevalence (subanalysis; 6 waves, 2018–2024; *n* = 21,216). Descriptive statistics for WC and AO are presented in Table S5; regression estimates for BMI/obesity and WC/AO are presented in Tables S6 and S7, respectively.

**Figure 4:**
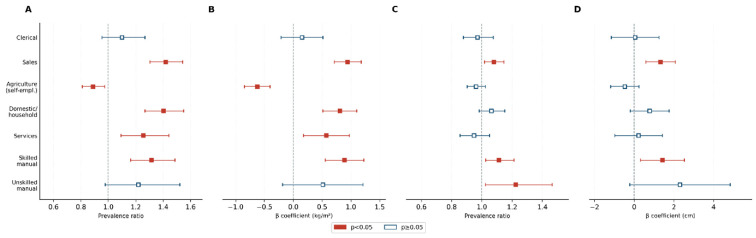
Crude estimates for adiposity outcomes by occupational group. Panel (**A**): Obesity (BMI ≥ 30), PR. Panel (**B**): BMI, β (kg/m^2^). Panel (**C**): Abdominal obesity (waist ≥ 88 cm), PR. Panel (**D**): Waist circumference, β (cm). Unadjusted models. Red square: *p* < 0.05; open blue square: *p* ≥ 0.05.

Models for obesity and BMI are presented in Figure 4 and Table S6. Overall mean BMI was 27.1 kg/m^2^, and obesity prevalence was 20.3%. Sales workers (PR 1.42; 95% CI 1.30–1.54), domestic/household workers (PR 1.40; 95% CI 1.27–1.55), and skilled manual workers (PR 1.31; 95% CI 1.16–1.49) had the highest prevalence ratios for obesity. Agricultural workers had lower obesity prevalence (PR 0.89; 95% CI 0.81–0.97) and the only significant decrease in BMI (β −0.62 kg/m^2^; 95% CI −0.85 to −0.40).

In the WC subanalysis, overall mean WC was 89.6 cm (SD 11.7) and AO prevalence was 53.6%. The pattern was partly consistent with BMI: sales (PR 1.08; 95% CI 1.02–1.15) and skilled manual work (PR 1.11; 95% CI 1.02–1.21) had the highest PRs for AO. The unskilled manual group showed a significant crude PR for AO (PR 1.22; 95% CI 1.02–1.46), a finding that was not statistically significant in the BMI analysis, suggesting that part of the adiposity in this group may be expressed centrally. Models for the WC subanalysis are presented in Table S7.

### 3.5. Secondary and Sensitivity Analyses

Secondary adjusted models are presented in Tables S8 and S9. Adjustment for age group, survey year, education, wealth, residence, natural region, and marital status attenuated several crude behavioral contrasts, especially in agriculture. Nevertheless, agricultural workers still had lower television viewing (adjusted PR [aPR] 0.86; 95% CI 0.81–0.92), current smoking (aPR 0.54; 95% CI 0.38–0.77), current alcohol use (aPR 0.85; 95% CI 0.77–0.93), and heavy alcohol use (aPR 0.36; 95% CI 0.22–0.58) than the reference group. Insufficient fruit and vegetable intake remained without a meaningful adjusted occupational gradient.

For adiposity, the adjusted models showed that obesity remained higher among clerical/administrative workers (aPR 1.16; 95% CI 1.01–1.33), sales workers (aPR 1.35; 95% CI 1.23–1.48), domestic/household workers (aPR 1.34; 95% CI 1.20–1.50), services workers (aPR 1.22; 95% CI 1.06–1.41), and skilled manual workers (aPR 1.25; 95% CI 1.10–1.43). The lower crude obesity estimate observed in agriculture did not persist after adjustment (aPR 1.02; 95% CI 0.91–1.15). For central adiposity, the adjusted AO estimates remained elevated but were more modest in sales (aPR 1.06; 95% CI 1.00–1.13) and skilled manual work (aPR 1.09; 95% CI 1.00–1.18), whereas the unskilled manual estimate became less precise (aPR 1.17; 95% CI 0.97–1.42).

Exploratory occupation-by-period interaction tests suggested some temporal heterogeneity for television viewing (*p* = 0.017) and obesity (*p* = 0.012), whereas other outcomes showed no strong evidence of change in occupational patterns across broad periods (Table S12). Missing-data sensitivity analyses showed that women excluded from the BMI analytic sample were younger and largely represented pregnancy-related anthropometric exclusions; WC exclusions were few but somewhat more urban and higher-income than included women (Tables S10 and S11).

## 4. Discussion

### 4.1. Main Findings

This study shows that occupational group is not a peripheral descriptor, but rather a substantive axis of health heterogeneity among Peruvian working women. Crude differences across groups were considerable in magnitude, and secondary adjusted analyses showed that part of the behavioral contrast—especially in agriculture—was explained by sociodemographic composition. In contrast, several adiposity contrasts persisted after adjustment, particularly among sales, domestic/household, services, and skilled manual workers. Thus, occupational group captures both compositional inequalities and occupation-linked patterns that remain relevant after accounting for major observed sociodemographic characteristics.

An equally important finding is that lifestyle and adiposity profiles were not concordant with one another and did not follow a simple gradient from occupations presumably more advantaged to those more disadvantaged. The professional/technical/managerial group showed the highest crude prevalences of current and heavy alcohol use, whereas the agricultural group showed fewer adverse behaviors without exhibiting equivalent anthropometric protection after adjustment. Likewise, insufficient fruit and vegetable intake was high in all groups. The WC subanalysis adds a relevant dimension: unskilled manual workers showed higher crude AO, but this estimate became less precise after adjustment. This suggests that body fat distribution may diverge according to type of work, while also indicating that central-adiposity signals in small occupational groups require cautious interpretation. Taken together, this reinforces the idea that the health of working women cannot be reduced to a single indicator or to a one-dimensional reading of social position.

### 4.2. Comparison with Other Studies

The lower crude prevalence of frequent television viewing and several tobacco- and alcohol-related outcomes among agricultural workers is consistent with studies linking greater occupational physical activity or less sitting time with more favorable behavioral profiles [5,10,11,15]. However, this pattern should not be interpreted as evidence that agricultural work is metabolically protective. Occupational physical activity is only one component of total energy balance and may coexist with poverty, food insecurity, irregular meals, limited dietary diversity, early-life nutritional deprivation, and fatigue that reduces recovery or leisure-time activity. The adjusted models reinforce this caution: the lower crude obesity estimate in agriculture disappeared after accounting for sociodemographic composition. Thus, the discordance observed in agricultural workers—fewer adverse behaviors as measured here, but no proportional protection in adiposity—should be understood as a hypothesis-generating finding rather than as evidence of a simple protective occupational effect.

The pattern of greater adiposity in sales, domestic/household occupations, services, and skilled manual work converges with the literature describing working women as a population exposed to a combination of occupational risk and unpaid burden [7,8]. The systematic review by Idris et al. identified overweight/obesity, low physical activity, and unhealthy diet as frequent problems among working women, with double burden and stress as recurrent mechanisms [7]. Da Silva et al. showed among Brazilian female shift workers that job stress was associated with a greater likelihood of obesity [8]. Our results extend this literature by showing that these inequalities can be observed at the population level in feminized occupations and in sectors with high relational or commercial intensity.

The occupational distribution of smoking and alcohol is consistent with the idea that these behaviors respond to specific work configurations rather than to a one-dimensional social hierarchy. Kava et al. documented in the United States that tobacco use varied according to unstable schedules, rotating shifts, and long working hours, in addition to occupational group [16]. Pérez-Romero et al. showed in Spain that heavy alcohol use by occupation differs between weekdays and weekends, with marked increases in higher-skilled occupations among women [17]. Our findings are compatible with the idea that sector-specific social norms, timing of consumption, and work-related sociability may be as relevant as occupational level itself.

The absence of clear occupational differences for insufficient fruit and vegetable intake likely reflects both a measurement limitation and a cross-cutting public health problem. The five-servings threshold is useful for surveillance and international comparability, but in this sample, it produced a near-ceiling pattern, with insufficient intake exceeding 87% in every occupational group. Therefore, this indicator should not be interpreted as evidence that diet is unrelated to occupation; rather, it suggests that the available measure was too coarse to capture differences in overall diet quality, meal timing, ultra-processed food consumption, or food access by occupation. Zaganjor et al. found differences in diet quality by occupation among employed women using a broader dietary index [11], supporting the need for more sensitive dietary measures in future surveys.

The relevance of the adiposity findings increases when considering body fat distribution. BMI and WC should not be interpreted as interchangeable indicators: BMI summarizes body mass relative to height, whereas WC better approximates central fat accumulation and may capture cardiometabolic risk not reflected by total body mass alone [4]. In the WC subanalysis, unskilled manual workers showed a significant crude excess of AO despite not showing a statistically significant excess of BMI-defined obesity. After adjustment, this estimate attenuated and became less precise, whereas sales and skilled manual work retained more consistent central-adiposity signals. This divergence suggests that some occupational groups may accumulate cardiometabolic risk through central adiposity even when overall BMI differences are modest or imprecisely estimated. The finding should nevertheless be interpreted cautiously because the unskilled manual group was small and WC was available only from 2018 onward.

### 4.3. Public Health Implications

The first implication is conceptual: occupation should be treated as a risk stratifier in the surveillance of noncommunicable diseases among women, not as a residual covariate. Global frameworks to promote physical activity insist on the need to intervene across all living environments [2,3], but our findings suggest that universal strategies without occupational segmentation will be insufficient.

The second implication is programmatic. Sectors do not require the same type of intervention. In services, offices, and occupations with greater screen exposure or work-related sociability, reducing sedentary time, modifying alcohol-consumption norms, and improving opportunities for physical activity during the workday may be priorities. For sales, domestic/household occupations, and manual work, the results point toward adiposity prevention that is sensitive to meal breaks, schedule regularity, access to healthy foods, and recovery time, rather than assuming that the movement inherent to work is sufficient to protect against obesity. The evidence of abdominal adiposity in unskilled manual work is a specific warning signal for this group, which combines high physical demands with unfavorable material conditions.

The third implication is gender-related. Occupation may operate through structural determinants and behavioral pathways that are analytically distinct. Structural determinants include gendered occupational segmentation, income constraints, informality, schedule predictability, access to social protection, and unpaid domestic or caregiving work. Behavioral pathways include eating routines, leisure-time screen exposure, alcohol and tobacco norms, and opportunities for physical activity or recovery. The present study directly measured occupational group and selected behaviors, but not job stress, working hours, childcare burden, or unpaid work. Therefore, these mechanisms should be interpreted as plausible explanations supported by prior literature, not as mechanisms empirically tested in this analysis.

The fourth implication concerns surveillance and applied research. If occupation is confirmed as a consistent marker of inequality in lifestyle behaviors and adiposity, national surveys should more precisely capture physical workload, working hours, shift work, informality, control over time, and more robust dietary measures. Future studies could also use clustering or latent-profile methods to evaluate joint lifestyle-adiposity phenotypes, provided that more granular dietary, physical-activity, and occupational-condition measures are available. Without these variables, occupation functions as a useful but incomplete signal for intervention.

### 4.4. Limitations

This study has important limitations. First, its repeated cross-sectional design does not allow temporal directionality or causal inference; some women may select into or leave certain jobs based on their health status or other unobserved factors, including a potential healthy-worker or survivor effect. Second, occupational group came from a broad standardized classification and did not allow detailed distinction of within-category heterogeneity in physical demands, hours worked, informality, night work, job stability, job control, psychosocial stress, commuting time, or unpaid domestic burden. This limitation is particularly relevant in settings where labor informality is common and may shape both working conditions and access to preventive resources. Third, several behavioral outcomes were self-reported and may be affected by recall or social desirability bias; frequency of television viewing is a proxy for leisure-time screen exposure rather than total sedentary time, and it may misclassify sedentary burden across occupations with different levels of occupational sitting. Similarly, the fruit-and-vegetable indicator is a highly summarized measure of diet and showed limited variation because insufficient intake was highly prevalent in all groups. Fourth, crude prevalence ratios were retained as the primary estimates; although this decision is consistent with the descriptive objective, the observed differences may be confounded by the unequal distribution of age, education, wealth, residence, region, marital status, reproductive history, and childcare burden across occupational groups. The secondary adjusted analyses reduce, but do not eliminate, this concern because they cannot account for unmeasured occupational and reproductive factors. Fifth, WC and AO were available only from 2018 onward, so these outcomes have lower temporal representativeness than BMI and should be interpreted as a subanalysis of central adiposity. Sixth, missing data were handled through complete-case analysis; missingness comparisons did not indicate a need for imputation, but they showed some differences between included and excluded women, especially for the WC subsample. Finally, the unskilled manual work group was small, and its estimates should be interpreted cautiously because of greater imprecision, and the analysis was restricted to women aged 18–49 years selected for the DHS/ENDES women’s questionnaire and therefore does not describe occupational adiposity patterns among older working women.

## 5. Conclusions

Among Peruvian working women aged 18–49 years, occupation identifies relevant disparities in lifestyle behaviors and adiposity levels. Self-employed agriculture showed the most favorable crude lifestyle profile—with the lowest observed prevalences of smoking, alcohol use, and television exposure—whereas the greatest burden of obesity and higher BMI was concentrated in sales, domestic/household occupations, services, and skilled manual work. Secondary adjusted analyses showed that several adiposity contrasts persisted after accounting for major sociodemographic characteristics, whereas some behavioral contrasts were attenuated, especially in agriculture. Insufficient fruit and vegetable intake was high in all groups. The WC subanalysis adds a clinically relevant signal: crude analyses identified excess AO in unskilled manual work not detectable through BMI, although this signal attenuated after adjustment and should be interpreted cautiously. Lifestyle and adiposity profiles did not follow a simple social gradient and were not concordant with one another, suggesting the simultaneous involvement of occupational, material, and gender-related mechanisms. We recommend incorporating occupation as a routine stratifier in national surveillance of noncommunicable diseases, developing differentiated interventions by occupational sector with a gender perspective, and strengthening longitudinal studies that can clarify mechanisms and causal trajectories.

## Figures and Tables

**Table 1:** Weighted characteristics of the primary analytic sample overall and by occupational group (*n* = 40,726).

Characteristic/ Level	Total (*n* = 40,726)	Prof./Tech./ Managerial (*n* = 6876)	Clerical/ Admin. (*n* = 2379)	Sales (*n* = 12,188)	Agric. (Self-Emp.) (*n* = 9805)	Domestic/ Household (*n* = 4225)	Services (*n* = 2054)	Skilled Manual (*n* = 2550)	Unskilled Manual (*n* = 649)
Sociodemographic Characteristics									
Age, mean (SD), years	31.7 (9.7)	32.9 (8.8)	31.1 (8.8)	31.7 (9.7)	31.7 (10.4)	30.7 (10.2)	30.6 (9.4)	31.6 (9.7)	31.4 (9.9)
Age group, %									
18–24 years	28.2	19.8	26.5	28.9	31.5	35.2	31.8	28.1	29.5
25–34 years	31.0	36.1	39.1	30.3	25.5	25.8	32.7	32.7	30.7
35–49 years	40.7	44.1	34.4	40.8	42.9	39.0	35.5	39.2	39.9
Educational level, %									
No formal education	1.3	0.0	0.1	0.7	4.7	1.0	0.3	1.8	0.8
Primary	14.7	1.0	1.1	12.3	42.8	15.0	7.1	13.0	24.4
Secondary	44.4	14.5	28.4	54.3	46.0	62.3	55.2	56.1	57.3
Higher	39.6	84.4	70.4	32.7	6.5	21.7	37.4	29.1	17.5
Wealth quintile, %									
Poorest	17.3	2.2	1.6	8.9	66.6	10.4	7.2	13.4	16.6
Poorer	20.9	8.2	11.8	24.9	23.4	31.5	18.5	23.9	32.0
Middle	21.4	16.9	18.7	27.5	7.1	27.5	27.6	28.3	24.4
Richer	21.1	27.9	28.3	24.2	2.3	20.3	27.2	23.6	19.4
Richest	19.3	44.8	39.6	14.6	0.5	10.3	19.6	10.9	7.7
Area of residence, %									
Urban	79.5	95.1	95.4	88.6	24.2	90.1	91.3	86.2	76.3
Rural	20.5	4.9	4.6	11.4	75.8	9.9	8.7	13.8	23.7
Natural region, %									
Lima	35.6	50.5	52.8	36.2	0.9	42.1	45.4	39.3	30.5
Coast (excl. Lima)	24.2	21.2	22.7	26.9	19.2	27.9	25.2	23.9	36.2
Highlands	26.9	19.3	15.9	23.5	53.8	19.7	19.8	27.5	23.4
Jungle	13.4	8.9	8.6	13.3	26.1	10.4	9.6	9.3	9.8
Marital status, %									
Never married	28.1	28.0	31.5	27.0	25.9	33.6	25.7	28.1	25.8
Married/cohabiting	57.2	57.1	52.9	57.7	62.8	48.7	57.7	60.1	56.8
Formerly married	14.7	14.9	15.6	15.3	11.3	17.8	16.6	11.8	17.3

SD: standard deviation. Weighted using the study’s pooled survey weight (2014–2019 and 2021–2024 waves). Column sample sizes are unweighted counts.

## Data Availability

The data presented in this study are available in the INEI microdata repository at https://proyectos.inei.gob.pe/microdatos/ (accessed on 10 February 2026).

## Supplementary Materials

The following supporting information can be downloaded at https://www.mdpi.com/article/10.3390/healthcare14121763/s1. Figure S1: Flow diagram; Figure S2: Temporal trends in behavioral outcomes; Figure S3: Prevalence ratios for daily smoking and disaggregated alcohol use; Table S1: STROBE checklist; Table S2: Weighted prevalence of lifestyle behaviors by occupational group; Table S3: Crude disparities in lifestyle behaviors by occupational group; Table S4: Crude disparities in daily smoking and disaggregated alcohol use; Table S5: WC and AO by occupational group; Table S6: Crude disparities in obesity and BMI by occupational group; Table S7: Crude disparities in abdominal obesity and waist circumference by occupational group; Table S8: Adjusted disparities in lifestyle behaviors by occupational group; Table S9: Adjusted disparities in adiposity indicators by occupational group; Table S10: Missing-data sensitivity for the BMI analytic sample; Table S11: Missing-data sensitivity for the WC analytic sample; Table S12: Exploratory occupation-by-period interaction tests.
