# Supplementary material for: Occupational Disparities in Lifestyle Behaviors and Adiposity Levels Among Working Women in Peru: A Pooled Repeated Cross-Sectional Analysis of 10 Rounds of a National Health Survey
Source: Healthcare (Basel). 2026 Jun 18;14(12):1763. doi: 10.3390/healthcare14121763 (PMC13299575; doi:10.3390/healthcare14121763)

healthcare

Supplementary Materials

Occupational Disparities in Lifestyle Behaviors and Adiposity Levels among Working Women in Peru: A Pooled Repeated Cross-Sectional Analysis of 10 Rounds of a National Health Survey

Víctor Juan Vera-Ponce, Jhosmer Ballena-Caicedo, Fiorella E. Zuzunaga-Montoya

Table S1. STROBE checklist for cross-sectional studies.

**No.**	**Recommendation**	**Location in the manuscript**
TITLE AND ABSTRACT	TITLE AND ABSTRACT	TITLE AND ABSTRACT
1a	Indicate the study design in the title or the abstract using a commonly used term	Title: "pooled repeated cross-sectional analysis of 10 rounds of a national health survey." Abstract, Methods: "Repeated cross-sectional analysis of ten DHS rounds from 2014–2019 and 2021–2024"
1b	Provide in the abstract an informative and balanced summary of what was done and what was found	Abstract includes: Background, Objective, Methods (design, population, exposure, outcomes, statistical approach), Findings (main prevalence ratios and effect sizes for lifestyle behaviors and adiposity), and Conclusions
INTRODUCTION	INTRODUCTION	INTRODUCTION
2	Background/rationale: Explain the scientific background and rationale for the investigation	Introduction, paragraphs 1–4: global burden of NCDs and adiposity in working-age women; non-linear relationship between work and health (sedentary behavior, occupational psychosocial risks); occupational gender segregation and double burden; evidence on diet quality by occupation and evidence gaps in Peru
3	Objectives: State-specific objectives, including any pre-specified hypotheses	Introduction, final paragraph: "to evaluate occupational disparities in lifestyle behaviors and adiposity among Peruvian working women"; Abstract, Objective section

METHODS	METHODS	METHODS
4	Study design: Present key elements of study design early in the paper	Methods, Study design: observational, analytical cross-sectional study based on repeated cross-sectional series of the ENDES. Ten survey rounds (2014–2019 and 2021–2024); 2020 excluded due to COVID-19 operational disruption. Reported following STROBE guidelines
5	Setting: Describe the setting, locations, and relevant dates	Methods, Data source: nationally representative household survey of Peru (ENDES/DHS), conducted by INEI. Stratified, multistage, complex sample design. Waves 2014–2019 and 2021–2024; Results, Analytic sample derivation: sample sizes by analytic component
6	Participants: Give the eligibility criteria and the sources and methods of selection	Methods, Study population and eligibility: women aged 18–49 years, usual residents of private households; linkable individual questionnaire + health module; currently working with a classified occupational group. Exclusion of "not working" category justified. Separate anthropometric samples: n=39,212 for BMI (all 10 waves); n=21,216 for waist circumference (6 waves, 2018–2024). Flow diagram in Supplementary Figure S1
7	Variables: Clearly define all outcomes, exposures, potential confounders, and effect modifiers	Methods, Main exposure: occupational group (8 active categories from DHS standard classification; reference = professional/technical/managerial). Methods, Outcomes: 5 lifestyle behaviors (binary); BMI and obesity (primary adiposity, 10 waves); waist circumference and abdominal obesity ≥88 cm (subanalysis, 6 waves). Methods, Covariates: age group, education, wealth quintile, area of residence, natural region, marital status, survey year
8	Data sources/measurement: For each variable, describe data sources and details of methods of assessment	Methods, Data source: individual women's questionnaire (occupational group, sociodemographic covariates) and health module (lifestyle behaviors, anthropometric measurements). Methods, Data preparation:

		plausibility ranges applied (weight 20–250 kg; height 120–210 cm; WC 45–180 cm; BMI 12–70 kg/m²). Education harmonization: QS25N as primary source; V106 (household questionnaire) as backup for 2015–2018 rounds
9	Bias: Describe any efforts to address potential sources of bias	Methods, Statistical analysis: primary crude models justified because the objective is descriptive and not causal; secondary adjusted models included age group, survey year, education, wealth quintile, residence, natural region, and marital status. Discussion, Limitations: selection/survivor bias, residual confounding, and measurement limitations discussed.
10	Study size: Explain how the study size was arrived at	Methods, Study population: the analytical sample was determined by the data available in all 10 ENDES waves meeting eligibility criteria; no formal sample size calculation was conducted as this is an analysis of secondary data. Final samples: behavioral n=40,726; anthropometric (BMI) n=39,212; WC subanalysis n=21,216
11	Quantitative variables: Explain how quantitative variables were handled	Methods, Outcomes: BMI and waist circumference analyzed as continuous outcomes (WLS regression, β coefficients). Methods, Data preparation: plausibility cutoffs applied. Obesity (BMI ≥30 kg/m²) and abdominal obesity (WC ≥88 cm) defined as binary outcomes for Poisson regression. Age modeled in prespecified categories
12a	Statistical methods: Describe all statistical methods, including those used to control for confounding	Methods, Statistical analysis: modified weighted Poisson regression (link log) with robust sandwich SE clustered at PSU level for binary outcomes (prevalence ratios); weighted linear regression with cluster-robust SE for continuous outcomes (β coefficients). Crude (unadjusted) models — rationale provided (descriptive objective; potential mediators among available covariates). Secondary adjusted models and exploratory temporal

		interaction tests are described in the Statistical Analysis section.
12b	Describe any methods used to examine subgroups and interactions	Methods, Statistical analysis: waist circumference outcomes analyzed as a pre-planned subanalysis restricted to 6 waves (2018–2024) due to measurement availability. Separate pooled weights calculated (weight/6 for WC; weight/10 for primary analysis). Exploratory occupation-by-period interaction tests were estimated for selected outcomes and reported in Table S12
12c	Explain how missing data were addressed	Methods, Statistical analysis: complete case analysis at the level of each outcome family. Multiple imputation not applied (missing data mainly from non-collected field measurements, not partial omissions). After education harmonization, missing data in main covariates was low. No correction for multiple comparisons
12d	If applicable, describe analytical methods taking account of sampling strategy	Methods, Statistical analysis: all analyses incorporated ENDES survey weights. Pooled weights rescaled by dividing by number of waves (10 or 6) to represent average population structure. Cluster-robust sandwich SE (Liang-Zeger with M/(M−1) correction) at PSU level. Data cleaning in Stata; models and figures generated in Python
12e	Describe any sensitivity analyses	Supplementary Figure S3 and Table S4: disaggregated alcohol use analysis (moderate drinking vs non-drinkers; excess drinking vs non-drinkers) as sensitivity/supplementary models. WC subanalysis (n=21,216) presented as planned sensitivity check for adiposity with a more specific measure of central adiposity
RESULTS	RESULTS	RESULTS
13a	Report the number of individuals at each stage of study	Results, Analytic sample derivation + Supplementary Figure S1 (flow diagram): total pooled records → women 18–49 years → primary waves → working women with

		occupational group → behavioral analytic sample (n=40,726) → anthropometric sample (n=39,212) → WC subsample (n=21,216)
13b	Give reasons for non-participation at each stage	Results, Analytic sample derivation; Supplementary Figure S1: 2020 excluded (COVID-19 operational disruption). "Not working" category excluded (heterogeneous group). Missing anthropometric measurements treated as exclusions for anthropometric analysis. Education harmonization allowed inclusion of 2015–2018 waves
14	Descriptive data: Give characteristics of study participants	Results, Sample characteristics; Table 1: weighted characteristics (age, education, wealth quintile, residence, natural region, marital status) by occupational group (n=40,726). Supplementary Figure 2 and Table S5 for WC/AO descriptives; Tables S6–S9 for crude and adjusted adiposity models
15	Outcome data: Report numbers of outcome events or summary measures	Results, Lifestyle behaviors (Figures 1, 3; Tables S1, S2). Results, Adiposity levels (Figures 2, 4; Tables S5–S9). Weighted prevalences and means with 95% CI reported for all outcomes by occupational group
16	Main results: Give unadjusted estimates and, if applicable, confounder-adjusted estimates	Results, Lifestyle behaviors: PR for all 5 behaviors by occupational group (Table S2); agriculture lowest PR for TV (0.49), smoking (0.14), and alcohol (0.39). Results, Adiposity levels: PR for obesity (Table S6); β for BMI (Table S6); PR for abdominal obesity and β for WC (Table S7). Primary estimates are unadjusted (crude) with 95% CI; secondary adjusted estimates are reported with 95% CI in Tables S8 and S9. Primary crude estimates and secondary adjusted estimates were reported; adjusted models are interpreted as conditional descriptive contrasts rather than causal effects
17	Other analyses: Report other analyses done—e.g., subgroups, interactions, sensitivity	Supplementary Figure S3 and Table S4: daily smoking and disaggregated alcohol use by occupational group.

		WC/abdominal obesity subanalysis (6 waves, 2018–2024; n=21,216) reported with explicit note on reduced temporal representativeness compared to primary analysis
DISCUSSION	DISCUSSION	DISCUSSION
18	Key results: Summarise key results with reference to study objectives	Discussion, Main findings (2 paragraphs): occupational heterogeneity in lifestyle behaviors and adiposity; agriculture most favorable lifestyle profile; sales, domestic/household, services and skilled manual highest adiposity burden; discordance between lifestyle and adiposity profiles; WC subanalysis identifies central adiposity signal in unskilled manual group
19	Limitations: Discuss limitations of the study, taking into account sources of potential bias or imprecision	Discussion, Limitations (6 items): (1) cross-sectional design — no temporal directionality or causal inference; selection/survivor bias possible; (2) broad occupational classification; (3) self-reported behavioral outcomes; TV as sedentary proxy; FV indicator as diet quality proxy; (4) crude estimates — potential confounding by differential sociodemographic distribution; (5) WC subanalysis—limited temporal representativeness (2018+); (6) small unskilled manual group (n=626 behavioral; n=229 WC)
20	Interpretation: Give a cautious overall interpretation of results considering objectives, limitations, multiplicity of analyses, and results from similar studies	Discussion, Comparison with other studies: comparison with literature on occupational PA and health (CESCAS, Korea), female shift workers (Brazil, Finland), tobacco/alcohol and work characteristics (Kava et al., USA; Pérez-Romero et al., Spain), diet quality (Zaganjor et al.), and adiposity distribution (Ross et al.; van den Berge et al.). Discussion, Public health implications (4 points)
21	Generalisability: Discuss the generalisability (external validity) of the study results	Discussion, Public health implications: findings from a nationally representative survey (ENDES) using 10 survey rounds; results represent working women aged 18–49 in Peru over 2014–2024. Limitations section notes that

		findings may not generalize beyond the occupational groups captured by the DHS standard classification
OTHER INFORMATION	OTHER INFORMATION	OTHER INFORMATION
22	Funding: Give the source of funding and the role of the funders	Declarations, Funding: article processing charges covered by the Vice-Rectorate for Research of the Toribio Rodríguez de Mendoza National University of Amazonas (UNTRM); no role in study design, data collection, analysis, publication decision, or manuscript preparation

Table S2. Weighted prevalence of lifestyle behaviors by occupational group.

**Behavioral outcome**	**Total (N=40,726)**	**Prof./tech./managerial (n=6,876)**	**Clerical/admin. (n=2,379)**	**Sales (n=12,188)**	**Agric. (self-emp.) (n=9,805)**	**Domestic/household (n=4,225)**	**Services (n=2,054)**	**Skilled manual (n=2,550)**	**Unskilled manual (n=649)**
Watching TV almost daily, %	55.4	62.1	64.6	59.8	30.7	59.1	61.2	56.4	57.7
Current smoking, %	4.6	6.8	8.3	4.5	1.0	4.9	5.8	3.7	2.1
Current alcohol use, %	32.8	43.4	42.5	32.8	16.8	31.7	39.5	29.5	26.0
Heavy alcohol use, %	2.3	4.4	3.1	1.9	0.8	2.0	2.4	1.6	1.2
<5 FV servings/day, %	90.6	90.1	89.1	90.9	92.6	90.3	87.8	90.7	90.3

FV: fruit and vegetables. Column n values are unweighted counts.

Table S3. Disparities in lifestyle behaviors by occupational group.

**Occupational group**	**TV almost daily PR (95% CI)**	**Current smoking PR (95% CI)**	**Current alcohol use PR (95% CI)**	**Heavy alcohol use PR (95% CI)**	**<5 FV servings/day PR (95% CI)**

Prof./tech./managerial (ref.)	1.00 (ref.)	1.00 (ref.)	1.00 (ref.)	1.00 (ref.)	1.00 (ref.)
Clerical/administrative	1.04 (0.99–1.10)	1.23 (0.94–1.60)	0.98 (0.90–1.07)	0.71 (0.47–1.07)	0.99 (0.96–1.01)
Sales	0.96 (0.93–1.00)	0.66 (0.54–0.81)	0.76 (0.71–0.80)	0.43 (0.33–0.57)	1.01 (0.99–1.03)
Agriculture (self-employed)	0.49 (0.47–0.52)	0.14 (0.10–0.19)	0.39 (0.36–0.42)	0.17 (0.12–0.27)	1.03 (1.01–1.04)
Domestic/household	0.95 (0.91–1.00)	0.72 (0.56–0.93)	0.73 (0.67–0.80)	0.45 (0.31–0.67)	1.00 (0.98–1.02)
Services	0.99 (0.93–1.05)	0.85 (0.63–1.14)	0.91 (0.82–1.00)	0.55 (0.35–0.87)	0.97 (0.95–1.00)
Skilled manual	0.91 (0.86–0.96)	0.54 (0.38–0.78)	0.68 (0.61–0.75)	0.37 (0.22–0.64)	1.01 (0.98–1.03)
Unskilled manual	0.93 (0.83–1.03)	0.30 (0.12–0.77)	0.60 (0.49–0.73)	0.27 (0.11–0.66)	1.00 (0.95–1.05)

Unadjusted models. Poisson regression with robust variance. Reference group: professional/technical/managerial. PR = prevalence ratio; 95% CI = 95% confidence interval.

Table S4. Disparities between occupational group and daily smoking and disaggregated alcohol use.

**Occupational group**	**Daily smoking PR (95% CI)**	**Moderate drinking vs non-drinkers PR (95% CI)**	**Heavy drinking vs non-drinkers PR (95% CI)**
Prof./tech./managerial (ref.)	1.00 (ref.)	1.00 (ref.)	1.00 (ref.)
Clerical/administrative	1.56 (0.78–3.13)	1.00 (0.91–1.09)	0.72 (0.48–1.07)
Sales	0.62 (0.35–1.09)	0.77 (0.72–0.82)	0.38 (0.29–0.50)
Agriculture (self-employed)	0.05 (0.01–0.15)	0.40 (0.36–0.43)	0.13 (0.08–0.19)
Domestic/household	0.62 (0.31–1.25)	0.74 (0.68–0.81)	0.39 (0.27–0.58)
Services	0.54 (0.21–1.37)	0.93 (0.84–1.03)	0.54 (0.34–0.83)
Skilled manual	0.43 (0.17–1.05)	0.69 (0.62–0.77)	0.32 (0.18–0.54)
Unskilled manual	0.07 (0.02–0.25)	0.61 (0.50–0.76)	0.22 (0.09–0.53)

Poisson regression with robust variance. Unadjusted models. PR = prevalence ratio; 95% CI = 95% confidence interval. Reference: professional/technical/managerial.

Table S5. WC and AO by occupational group.

Outcome	Total (N=21,216)	Prof./tech./managerial (n=3,769)	Clerical/admin. (n=1,132)	Sales (n=6,283)	Agric. (self-emp.) (n=4,860)	Domestic/household (n=2,245)	Services (n=1,171)	Skilled manual (n=1,527)	Unskilled manual (n=229)
Waist circumference, mean (SD), cm	89.6 (11.7)	89.0 (10.9)	89.0 (11.3)	90.3 (12.2)	88.5 (11.2)	89.8 (12.0)	89.2 (11.7)	90.4 (11.8)	91.3 (11.3)
Abdominal obesity (waist ≥88 cm), %	53.6	51.8	50.4	56.0	49.9	55.2	49.2	57.8	63.5

Subanalysis: WC available in DHS since 2018. Column n values are unweighted counts.

Table S6. Disparities in obesity and BMI by occupational group.

Occupational group	Obesity (BMI ≥30) PR (95% CI)	BMI β (95% CI), kg/m²
Prof./tech./managerial (ref.)	1.00 (ref.)	0.00 (ref.)
Clerical/administrative	1.10 (0.95–1.27)	0.15 (-0.21, 0.52)
Sales	1.42 (1.30–1.54)	0.94 (0.71, 1.18)
Agriculture (self-employed)	0.89 (0.81–0.97)	-0.62 (-0.85, -0.40)
Domestic/household	1.40 (1.27–1.55)	0.81 (0.51, 1.10)
Services	1.25 (1.09–1.44)	0.58 (0.18, 0.97)
Skilled manual	1.31 (1.16–1.49)	0.89 (0.56, 1.23)
Unskilled manual	1.22 (0.98–1.52)	0.51 (-0.19, 1.21)

Poisson regression with robust variance. Unadjusted models. PR = prevalence ratio; β = beta coefficient; 95% CI = 95% confidence interval. Reference: professional/technical/managerial.

Table S7. Disparities in abdominal obesity and waist circumference by occupational group.

Occupational group	Abdominal obesity (waist ≥88 cm) PR (95% CI)	Waist circumference β (95% CI), cm
Prof./tech./managerial (ref.)	1.00 (ref.)	0.00 (ref.)
Clerical/administrative	0.97 (0.88–1.07)	0.05 (-1.15, 1.26)
Sales	1.08 (1.02–1.15)	1.33 (0.59, 2.07)
Agriculture (self-employed)	0.96 (0.90–1.02)	-0.47 (-1.18, 0.24)

Domestic/household	1.06 (0.98–1.15)	0.78 (-0.20, 1.76)
Services	0.95 (0.86–1.05)	0.22 (-0.97, 1.42)
Skilled manual	1.11 (1.02–1.21)	1.42 (0.32, 2.53)
Unskilled manual	1.22 (1.02–1.46)	2.31 (-0.22, 4.84)

Poisson regression with robust variance. Unadjusted models. PR = prevalence ratio; β = beta coefficient; 95% CI = 95% confidence interval. Reference: professional/technical/managerial.

Table S8. Adjusted disparities in lifestyle behaviors by occupational group.

**Occupational group**	**TV almost daily aPR (95% CI)**	**Current smoking aPR (95% CI)**	**Current alcohol use aPR (95% CI)**	**Heavy alcohol use aPR (95% CI)**	**<5 FV servings/day aPR (95% CI)**
Professional/technical/managerial (ref.)	1.00 (ref.)	1.00 (ref.)	1.00 (ref.)	1.00 (ref.)	1.00 (ref.)
Clerical/administrative	1.01 (0.96–1.07)	1.18 (0.91–1.54)	1.03 (0.94–1.12)	0.73 (0.48–1.11)	0.99 (0.96–1.02)
Sales	1.05 (1.01–1.09)	0.92 (0.75–1.13)	0.99 (0.92–1.05)	0.62 (0.45–0.84)	1.00 (0.99–1.02)
Agriculture (self-employed)	0.86 (0.81–0.92)	0.54 (0.38–0.77)	0.85 (0.77–0.93)	0.36 (0.22–0.58)	1.00 (0.98–1.02)
Domestic/household	1.07 (1.02–1.13)	0.99 (0.75–1.30)	1.01 (0.92–1.10)	0.61 (0.40–0.93)	1.00 (0.98–1.02)
Services	1.05 (0.99–1.12)	1.04 (0.77–1.39)	1.12 (1.02–1.24)	0.73 (0.46–1.17)	0.97 (0.95–1.00)
Skilled manual	1.04 (0.98–1.10)	0.82 (0.57–1.17)	0.92 (0.83–1.02)	0.55 (0.31–0.99)	1.00 (0.97–1.02)
Unskilled manual	1.01 (0.91–1.13)	0.47 (0.19–1.20)	0.88 (0.72–1.09)	0.40 (0.16–1.00)	0.99 (0.94–1.04)

Adjusted models. Poisson regression with robust variance. Adjusted for age group, survey year, educational level, wealth quintile, area of residence, natural region, and marital status. Reference group: professional/technical/managerial. aPR = adjusted prevalence ratio; 95% CI = 95% confidence interval; FV = fruit and vegetables.

Table S9. Adjusted disparities in adiposity indicators by occupational group.

**Occupational group**	**Obesity (BMI ≥30) aPR (95% CI)**	**BMI β (95% CI), kg/m²**	**AO (WC ≥88 cm) aPR (95% CI)**	**WC β (95% CI), cm**
Professional/technical/managerial (ref.)	1.00 (ref.)	0.00 (ref.)	1.00 (ref.)	0.00 (ref.)
Clerical/administrative	1.16 (1.01–1.33)	0.40 (0.04–0.76)	1.03 (0.93–1.13)	0.88 (-0.25–2.00)
Sales	1.35 (1.23–1.48)	0.91 (0.66–1.16)	1.06 (1.00–1.13)	1.23 (0.47–2.00)

Agriculture (self-employed)	1.02 (0.91–1.15)	-0.01 (-0.29–0.27)	0.96 (0.89–1.03)	-0.50 (-1.38–0.39)
Domestic/household	1.34 (1.20–1.50)	0.86 (0.55–1.17)	1.05 (0.97–1.14)	0.72 (-0.27–1.71)
Services	1.22 (1.06–1.41)	0.64 (0.25–1.03)	0.97 (0.88–1.08)	0.64 (-0.51–1.79)
Skilled manual	1.25 (1.10–1.43)	0.85 (0.51–1.19)	1.09 (1.00–1.18)	1.18 (0.11–2.24)
Unskilled manual	1.18 (0.95–1.48)	0.54 (-0.14–1.21)	1.17 (0.97–1.42)	1.75 (-0.67–4.18)

Adjusted models. Poisson regression with robust variance was used for obesity and abdominal obesity; weighted linear regression was used for BMI and waist circumference. All models were adjusted for age group, survey year, educational level, wealth quintile, area of residence, natural region, and marital status. Reference group: professional/technical/managerial. aPR = adjusted prevalence ratio; β = beta coefficient; AO = abdominal obesity; WC = waist circumference; 95% CI = 95% confidence interval.

Table S10. Missing-data sensitivity for the BMI analytic sample.

**Section**	**Characteristic**	**Level**	**Included unweighted n**	**Included weighted %/mean**	**Excluded unweighted n**	**Excluded weighted %/mean**	**Weighted difference**
Sample	BMI analytic sample among behavioral primary sample	N	39212		1514		
Sociodemographic	Age	Mean (SD), years	39212	31.8 (9.7)	1514	29.3 (7.7)	2.5
Sociodemographic	Age group, %	15–24	9457	28.1	438	31.8	-3.7
Sociodemographic	Age group, %	25–34	15039	30.6	724	42.1	-11.5
Sociodemographic	Age group, %	35–49	14716	41.3	352	26.1	15.2
Sociodemographic	Education, %	No formal education	627	1.3	23	1.2	0.1
Sociodemographic	Education, %	Primary	7100	14.8	263	12.4	2.3
Sociodemographic	Education, %	Secondary	17326	44.5	660	43.8	0.7
Sociodemographic	Education, %	Higher	14159	39.5	568	42.6	-3.1
Sociodemographic	Wealth quintile, %	Poorest	10223	17.3	421	17.0	0.4
Sociodemographic	Wealth quintile, %	Poorer	10230	20.9	370	20.0	1.0
Sociodemographic	Wealth quintile, %	Middle	8026	21.3	311	23.4	-2.1
Sociodemographic	Wealth quintile, %	Richer	6256	21.1	227	20.6	0.5
Sociodemographic	Wealth quintile, %	Richest	4477	19.3	185	19.0	0.3

Sociodemographic	Residence, %	Urban	27192	79.5	1045	80.4	-0.9
Sociodemographic	Residence, %	Rural	12020	20.5	469	19.6	0.9
Sociodemographic	Natural region, %	Lima	4668	35.5	195	38.4	-2.9
Sociodemographic	Natural region, %	Coast (excluding Lima)	11083	24.2	434	22.7	1.5
Sociodemographic	Natural region, %	Highlands	14066	27.0	475	22.6	4.4
Sociodemographic	Natural region, %	Jungle	9395	13.3	410	16.2	-3.0
Sociodemographic	Marital status, %	Never married	10072	28.3	281	23.4	4.9
Sociodemographic	Marital status, %	Married/cohabiting	23231	56.8	1100	68.0	-11.2
Sociodemographic	Marital status, %	Formerly married	5909	15.0	133	8.6	6.3
Occupational	Occupational group, %	Professional/technical/managerial	6598	19.7	278	23.1	-3.4
Occupational	Occupational group, %	Clerical/administrative	2270	6.7	109	8.1	-1.4
Occupational	Occupational group, %	Sales	11759	30.9	429	29.2	1.7
Occupational	Occupational group, %	Agriculture (self-employed)	9438	16.8	367	15.9	0.9
Occupational	Occupational group, %	Domestic/household	4101	11.9	124	9.1	2.8
Occupational	Occupational group, %	Services	1976	5.6	78	5.3	0.3
Occupational	Occupational group, %	Skilled manual	2444	6.9	106	8.0	-1.2
Occupational	Occupational group, %	Unskilled manual	626	1.5	23	1.3	0.2
Survey	Survey year, %	2014	4142	10.4	191	14.8	-4.4
Survey	Survey year, %	2015	4769	11.4	194	10.2	1.2
Survey	Survey year, %	2016	4552	11.8	209	12.0	-0.2
Survey	Survey year, %	2017	4309	10.9	170	12.1	-1.2
Survey	Survey year, %	2018	3735	9.3	143	9.2	0.2

Survey	Survey year, %	2019	3562	9.2	125	7.5	1.7
Survey	Survey year, %	2021	2598	7.0	99	6.4	0.6
Survey	Survey year, %	2022	3233	8.8	107	8.3	0.5
Survey	Survey year, %	2023	3952	10.1	140	10.0	0.1
Survey	Survey year, %	2024	4360	11.2	136	9.6	1.7
Anthropometric eligibility	Pregnancy status, %	Not pregnant	39212	100.0	263	24.4	75.6
Anthropometric eligibility	Pregnancy status, %	Pregnant	0	0.0	1251	75.6	-75.6

Values are unweighted counts and weighted percentages or means, as applicable. Weighted difference is calculated as included minus excluded.

Table S11. Missing-data sensitivity for the WC analytic sample.

**Section**	**Characteristic**	**Level**	**Included unweighted n**	**Included weighted %/mean**	**Excluded unweighted n**	**Excluded weighted %/mean**	**Weighted difference**
Sample	WC analytic sample among non-pregnant behavioral sample in WC years	N	21216		392		
Sociodemographic	Age	Mean (SD), years	21216	31.9 (9.7)	392	32.8 (9.7)	-0.9
Sociodemographic	Age group, %	18–24	5025	27.2	98	23.8	3.4
Sociodemographic	Age group, %	25–34	8467	31.7	141	32.1	-0.5
Sociodemographic	Age group, %	35–49	7724	41.2	153	44.1	-2.9
Sociodemographic	Education, %	No formal education	281	1.0	6	0.9	0.1
Sociodemographic	Education, %	Primary	3147	10.8	42	5.7	5.1
Sociodemographic	Education, %	Secondary	9516	44.5	160	40.3	4.2
Sociodemographic	Education, %	Higher	8272	43.7	184	53.2	-9.5
Sociodemographic	Wealth quintile, %	Poorest	5805	15.2	83	8.3	6.9
Sociodemographic	Wealth quintile, %	Poorer	5506	20.1	83	15.4	4.8
Sociodemographic	Wealth quintile, %	Middle	4286	22.3	63	15.6	6.7

Sociodemographic	Wealth quintile, %	Richer	3300	22.0	87	29.1	-7.1
Sociodemographic	Wealth quintile, %	Richest	2319	20.4	76	31.6	-11.2
Sociodemographic	Residence, %	Urban	14787	84.5	302	92.3	-7.8
Sociodemographic	Residence, %	Rural	6429	15.5	90	7.7	7.8
Sociodemographic	Natural region, %	Lima	2621	40.0	82	57.1	-17.1
Sociodemographic	Natural region, %	Coast (excluding Lima)	5966	24.2	118	19.2	5.0
Sociodemographic	Natural region, %	Highlands	7472	23.5	128	18.0	5.5
Sociodemographic	Natural region, %	Jungle	5157	12.3	64	5.7	6.6
Sociodemographic	Marital status, %	Never married	5164	26.8	100	27.6	-0.8
Sociodemographic	Marital status, %	Married/cohabiting	12511	56.5	247	57.3	-0.9
Sociodemographic	Marital status, %	Formerly married	3541	16.7	45	15.0	1.7
Occupational	Occupational group, %	Professional/technical/managerial	3769	21.2	96	32.3	-11.1
Occupational	Occupational group, %	Clerical/administrative	1132	6.2	26	8.9	-2.7
Occupational	Occupational group, %	Sales	6283	31.8	116	29.7	2.1
Occupational	Occupational group, %	Agriculture (self-employed)	4860	13.3	65	6.1	7.2
Occupational	Occupational group, %	Domestic/household	2245	12.3	38	9.7	2.7
Occupational	Occupational group, %	Services	1171	6.4	24	5.9	0.5
Occupational	Occupational group, %	Skilled manual	1527	7.7	26	7.3	0.4
Occupational	Occupational group, %	Unskilled manual	229	1.0	1	0.2	0.9
Survey	Survey year, %	2018	3673	16.5	71	26.0	-9.4
Survey	Survey year, %	2019	3484	16.3	78	17.7	-1.4
Survey	Survey year, %	2021	2577	12.6	39	10.1	2.4

Survey	Survey year, %	2022	3211	15.9	53	11.4	4.5
Survey	Survey year, %	2023	3934	18.3	78	17.6	0.7
Survey	Survey year, %	2024	4337	20.4	73	17.2	3.2
Anthropometric eligibility	Pregnancy status, %	Not pregnant	21216	100.0	392	100.0	0.0

Values are unweighted counts and weighted percentages or means, as applicable. Weighted difference is calculated as included minus excluded.

Table S12. Exploratory occupation-by-period interaction tests.

Outcome	Survey periods compared	Global interaction p value	Interpretation
TV watching almost every day	2014–2016; 2017–2019; 2021–2024	0.017	Evidence of temporal heterogeneity
Current smoking	2014–2016; 2017–2019; 2021–2024	0.065	Borderline; no strong evidence after cautious interpretation
Current alcohol use	2014–2016; 2017–2019; 2021–2024	0.178	No strong evidence of temporal heterogeneity
Obesity (BMI ≥30)	2014–2016; 2017–2019; 2021–2024	0.012	Evidence of temporal heterogeneity
Body mass index	2014–2016; 2017–2019; 2021–2024	0.097	No strong evidence of temporal heterogeneity
Abdominal obesity (WC ≥88 cm)	2018–2019; 2021–2024	0.056	Borderline; no strong evidence after cautious interpretation
Waist circumference	2018–2019; 2021–2024	0.304	No strong evidence of temporal heterogeneity

P values correspond to global occupation-by-period interaction tests in weighted models. These analyses were exploratory and are not interpreted as causal time trends.

Figure S1. Flow diagram. DHS Peru, 2014–2019 and 2021–2024. The anthropometric sample was restricted to observations with available BMI after excluding pregnant women.

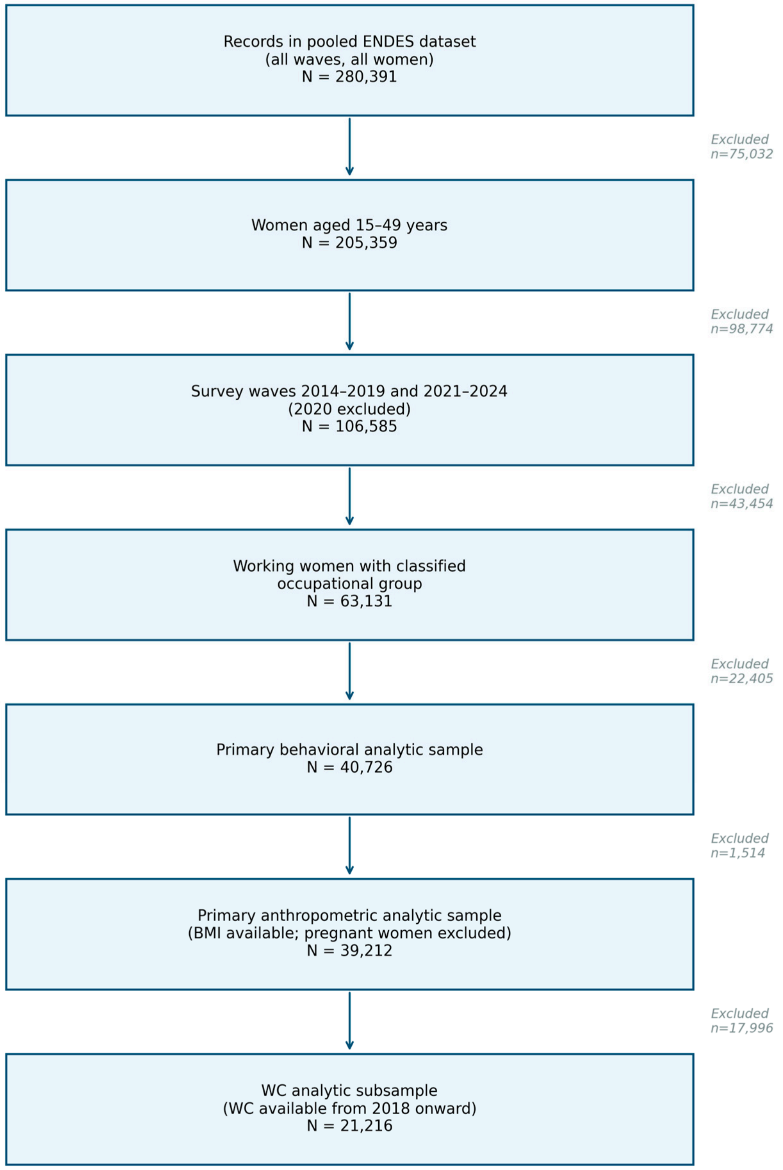

Figure S2. Temporal trends in behavioral outcomes by survey year. Vertical axes use different scales according to the range of each outcome.

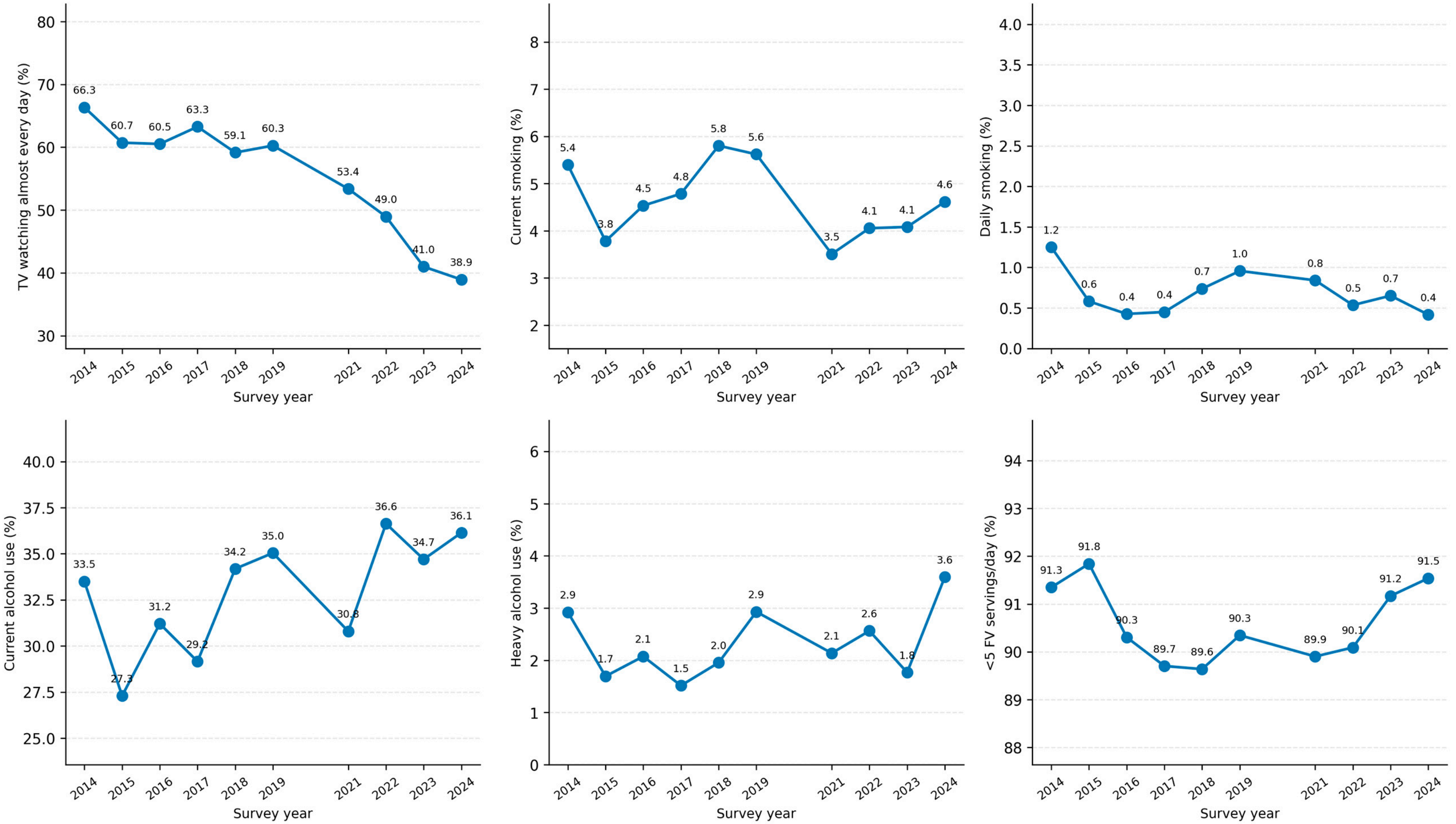

Figure S3. Prevalence ratios for daily smoking and disaggregated alcohol use. Reference: professional/technical/managerial. Unadjusted models.

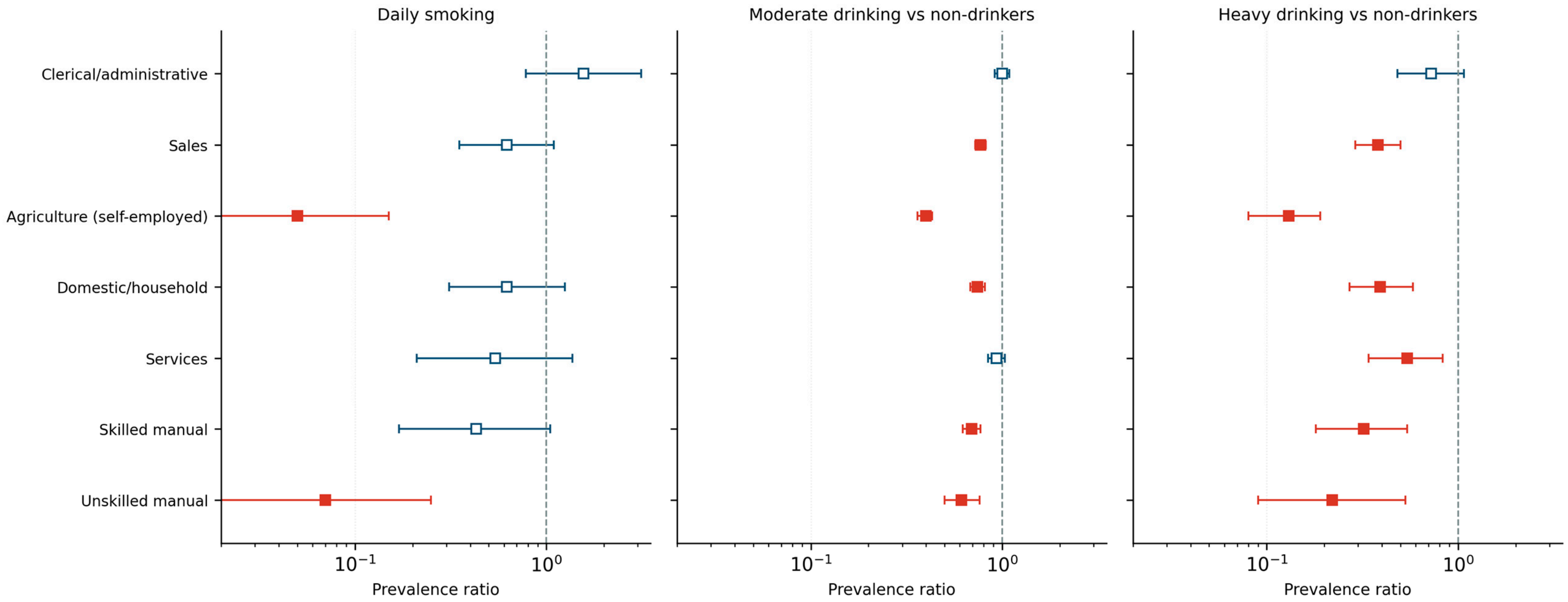

Supplement: Supplementary file 1 [file healthcare-14-01763-s001.zip › healthcare-4290048-supplementary.pdf]
